# Complex Sex Differences in Life Expectancy in French Guiana

**DOI:** 10.3390/ijerph20136195

**Published:** 2023-06-21

**Authors:** Mathieu Nacher, Célia Basurko, Laure Manuella Imounga, Qiannan Wang, Astrid Van Melle, Aude Lucarelli, Antoine Adenis, Kinan Drak Alsibai, Najeh Hcini, Nadia Sabbah

**Affiliations:** 1 CIC INSERM 1424, Centre Hospitalier de Cayenne, Cayenne 97300, French Guiana; 2 Département Formation Recherche Santé, Université de Guyane, Cayenne 97300, French Guiana; 3 Amazonian Infrastructures for Population Health, Cayenne 97300, French Guiana; 4 Registre des Cancers de Guyane, Cayenne 97300, French Guiana; 5 Département Recherche Innovation Santé Publique, Centre Hospitalier de Cayenne, Cayenne 97300, French Guiana; 6 Centre de Ressources Biologiques Amazonie, Centre Hospitalier de Cayenne, Cayenne 97300, French Guiana; 7 Service d’Anatomopathologie, Centre Hospitalier de Cayenne, Cayenne 97300, French Guiana; 8 Western French Guiana Hospital, Saint Laurent du Maroni 97320, French Guiana; 9 Service d’Endocrinologie Diabétologie, Centre Hospitalier de Cayenne, Cayenne 97300, French Guiana

**Keywords:** life expectancy, sex differences, causes of death, environment, French Guiana

## Abstract

In the complex context of French Guiana, different vulnerabilities and different risk factors between genders may lead to complex differences in health outcomes, mortality, and life expectancy. Our aim was, thus, to compare male and female mortality and life expectancy, to compare it between French Guiana and mainland France, and to look at temporal trends and the main specific causes of death in order to identify actionable singularities. National databases were used to obtain life expectancy at birth, at 20, 40, and 60 years, and mortality statistics. Standardized death rates and causes of death for French Guiana and mainland France were obtained through the CEPIDC, which analyzes information from death certificates. When comparing with mainland France, life expectancy at birth was significantly shorter both in males and females (mean = −2.9 years); life expectancy at 20 years, which allows to remove the effect of the greater child mortality in French Guiana, was also shorter in French Guiana for males (mean = −1.8 years) and females (mean = −2 years). The differences between mainland France and French Guiana regarding life expectancy at 40 and 60 years (mean = −1.5 and −1.3 years) was mainly found among females, males in French Guiana life expectancy at 40 and 60 years was closer to that in mainland France (mean = −0.8 and −0.6 years). Although they have a greater life expectancy at birth than men, women in French Guiana are substantially more affected by overweight/obesity and type 2 diabetes. The observed patterns of life expectancy at different ages presumably reflect the burden of external causes and AIDS in males and perhaps metabolic diseases in women.

## 1. Introduction

Life expectancy at birth is notoriously shorter in men than in women. This was known since, for the first time, life tables differentiated men and women during the 18th century [1]. This is also a near ubiquitous finding since circumstances where female mortality exceed that of males becoming increasingly rare [2,3]. The observation of such a statistical regularity led to efforts to understand the phenomenon in order to adapt social policies and to improve public health. Overall, genetics, hormones, immunity, environments, social roles, and behaviors form complex combinations, resulting in the seemingly clearcut difference in life expectancy between sexes. Despite this, however, there is a paradox wherein women live longer than men at all ages, but spend a higher proportion of their total life in poorer health [4,5].

French Guiana is an overseas French territory located between Suriname and Brazil. It has an ethnically diverse population of circa 300,000 inhabitants living on a territory about the size of England, with over 90% covered by primary Amazonian forest. Population ancestral origins in French Guiana are diverse and include descendants of the African slave trade, Caucasians, Amerindians, Chinese, Hmong, Lao, and Middle Eastern people. Different ancestral populations may have different predispositions for different diseases: hence, African ancestry was associated with hypertension, obesity, and diabetes, for example [6]. There is no administrative statutory difference with mainland France. As in France, school is mandatory, roads and infrastructure are the authorities’ responsibility, French drivers licenses are mandatory, municipalities deliver French passports and ID cards, minimum wage is that of France and there is a 30% bonus (to compensate for costs due to the distance from France), most aspects of social life are in reference to France and Europe, not South America. After controlling for median age, there is no difference in health expenditure per capita between French Guiana and mainland France [7]. As in France, men and women have the same rights, most committees in various areas of society must follow a parity rule with equivalent numbers of men and women, and in higher education, young women often outperform men, for example in medical school, where over 75% of those admitted are women.

Most of the population lives in three cities on a coastal strip Cayenne, Kourou, and Saint Laurent du Maroni, but 20% live in the interior of French Guiana with no road access and, mostly, no specialized care but a network of primary care centers scattered in the remote villages. As with many countries, French Guiana transitioned from a high burden of infectious diseases and child mortality towards a greater burden of non-communicable diseases. French Guiana has the highest gross domestic product (GDP) per capita in Latin America and, by far, the greatest health expenditure per capita [8]. The health and the social security system are the universal French system where health is a basic right inscribed in the law of the land. Hence, the poorest benefit from the “Protection Universelle MAladie”, which covers them for public and private care; undocumented immigrants residing in French Guiana also benefit from the “Aide Medicale Etat”, which gives them similar benefits as the Protection Universelle MAladie. This is important because in French Guiana, 29% of the population, and nearly half of the adults, are immigrants, usually attracted by the territory’s economic prospects. Thus, French Guiana’s highest GDP per capita in Latin America tends to overshadow the fact that over half of its population lives under the poverty threshold [9]. Over 36% of families consist of single mothers, and 57% of unemployed persons are women. This results in complex social inequalities of health regarding infectious, nutritional, obstetrical, and non-communicable diseases. These inequalities often correspond to delays in diagnosis and access to care, and often lead to renouncing care altogether [10,11,12]. However, for prolonged diseases, such as diabetes or HIV, once persons are in care, the universal health system in French Guiana actually levels prognoses and erases the differences that are observed before patients are taken care of by the system [13]. We hypothesized that, in the complex context of French Guiana, different vulnerabilities and different risk factors between genders [14] may lead to complex differences in health outcomes, mortality, and life expectancy. Our aim was, thus, to compare male and female mortality and life expectancy, to compare it between French Guiana and mainland France, and to look at temporal trends and the main specific causes of death in order to identify actionable singularities.

## 2. Methods

### 2.1. Data Sources

INSEE (National Institute for Statistics and Economics Studies) databases were used to obtain life expectancy at birth, at 20, 40, and 60 years, and mortality statistics [9]. Standardized death rates and causes of death for French Guiana and mainland France were obtained through the CEPIDC, which analyzes information from death certificates for INSERM (National Institute for Health and Medical Research) [15]. All deaths on the territory require a death certificate by law. Processing of death certificates is a lengthy process; therefore, there is a 5-year lag (in 2022, the results are available until 2017), whereas INSEE data were available until 2021. These differences explain why the x axis date range varied depending on what data source was used. Indicators were compared between French Guiana and mainland France.

### 2.2. Statistical Methods

The INSEE conducts censuses to update the population count and structure in French Guiana. These censuses concern all persons residing on the territory, even undocumented immigrants. This population data constitutes the denominator for rate calculations by the CEPIDC.

Standardized mortality rates were standardized using the 2013 European Population.

For the temporal representation of death rates, linear regression lines were fitted for French Guiana because the small population led to noisy curves and jagged lines in comparison with those of mainland France resulting from greater numbers.

Data were plotted using Excel and STATA 16. Curves were smoothed using a moving average.

Finally, given the broad socioeconomic contrasts in French Guiana, crude measures can be misleading. It is, thus, likely that the richer half of the population living in French Guiana has a very different life expectancy than the poorer areas. The median income in French Guiana (920 euros) is only half of that of France (1837 euros) and food is 34% more expensive in French Guiana and large within territory; differences in life statistics are, thus, expectable. INSEE recently developed a tool to compute life expectancy at birth by income strata; we used this tool to compare the 1000 euro per month group and the 2000 euro per month group [16]. Given that 23% of persons live with less than 550 euros per month, we also used the INSEE tool to compare the difference in life expectancy at birth between those living with 500 euros per month versus those living with 2000 euros per month.

## 3. Results

**Figure 1:**
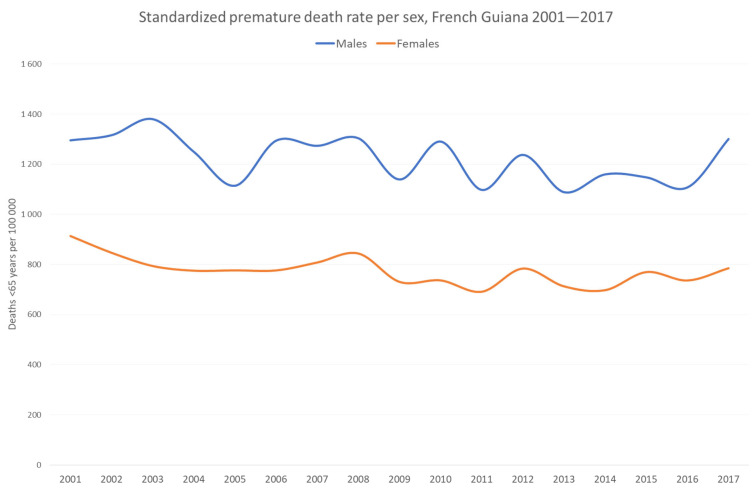
Comparison of yearly premature death by sex. Males are represented in blue, females in orange.

Figure 1 shows the standardized premature death rate per sex in French Guiana. Between 2001 and 2016, there was a gradual decline. In 2017, a prolonged blockade of the city and a 74-day hospital strike hampered access to care, which may have explained the surge in the premature death rate. The jagged lines correspond to the exacerbation of fluctuations due to the small population size of French Guiana.

**Figure 2:**
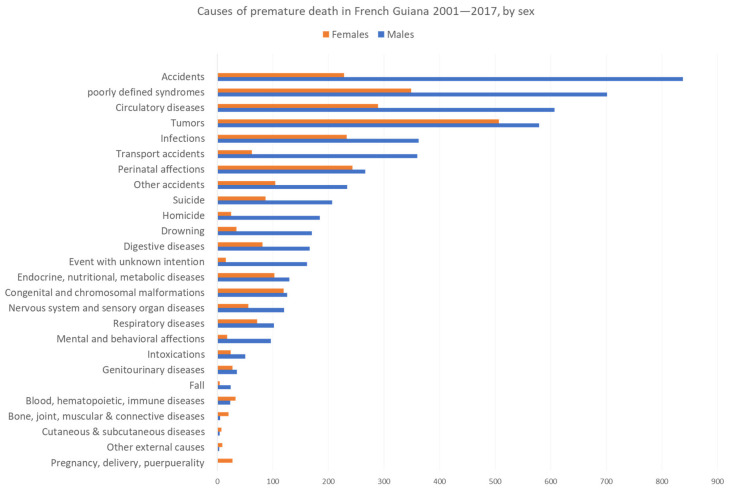
Cause of premature death in French Guiana 2001–2017, by sex. Males are represented in blue and females in orange.

**Figure 3:**
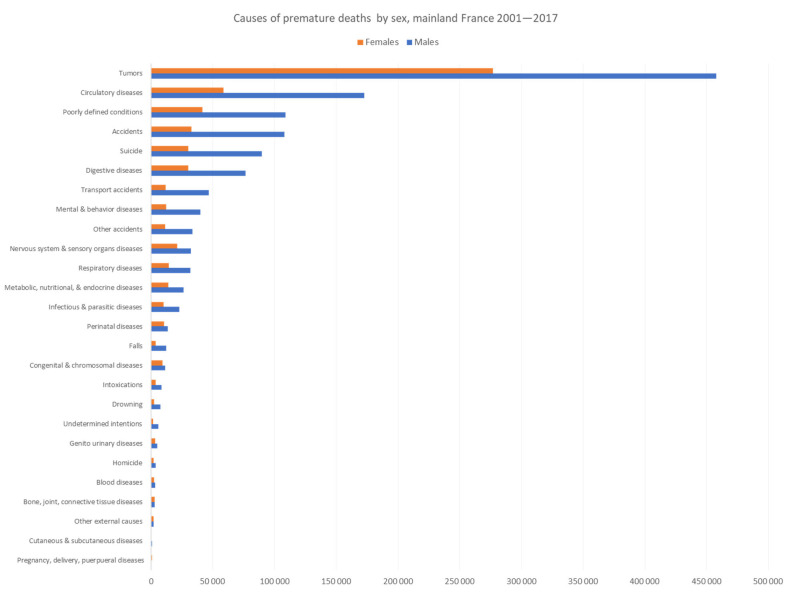
Causes of premature deaths by sex, mainland France 2001–2017. Males are represented in blue and females in orange.

Figure 2 and Figure 3 show the main causes of premature mortality by sex for French Guiana and mainland France, respectively. Data are sorted by main cause of death for males because they were found to have a greater premature mortality. The figures show, for males, substantial differences between French Guiana and mainland France with more accidents, more infections, more perinatal affections, more homicides, and more drownings in French Guiana than in mainland France where tumors, suicides, digestive diseases, and mental and behavioral problems were among the top 10 causes of premature death. For females, the ranking order was different, with tumors as first cause of premature death both in French Guiana and in mainland France. However, accidents, circulatory diseases, infections, perinatal affections, metabolic diseases, and congenital and chromosomal malformations seemed to be proportionally more important in French Guiana than mainland France. Homicides ranked among the top 10 causes of premature death in French Guiana. For homicides, the male bias was even greater in French Guiana, with over seven times the number of male homicides than female homicides versus 1.9 the number of male homicides than female homicides in mainland France; the more pronounced male bias in French Guiana was also apparent for drowning 5 versus 3.2 in mainland France.

By contrast, suicide ranked higher in mainland France than in French Guiana with a greater male bias than in French Guiana.

**Figure 4:**
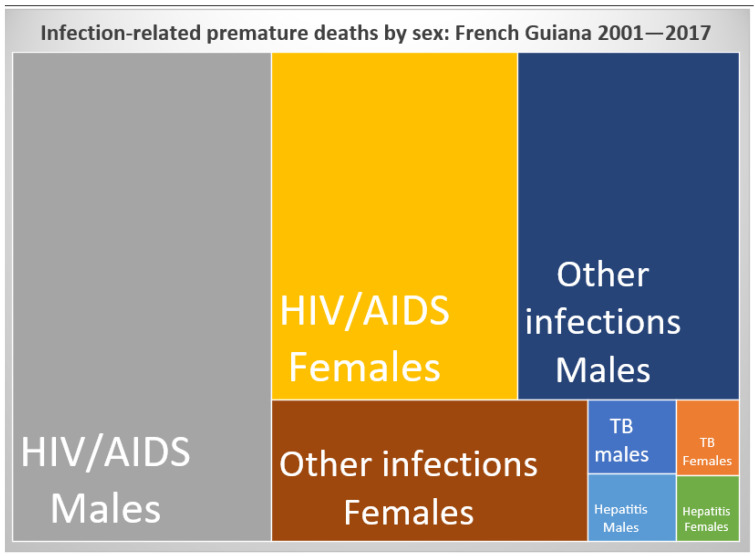
Evolution of the main causes of death. Rectangles are proportional in size to their respective importance in order to permit a visual appreciation of the burden of different pathologies. (TB stands for tuberculosis).

Figure 4 shows infections by sex and shows the preponderance of HIV-related deaths, notably among men. However, this excess mortality from infectious diseases in males also concerned tuberculosis, hepatitis, and other infections.

**Figure 5:**
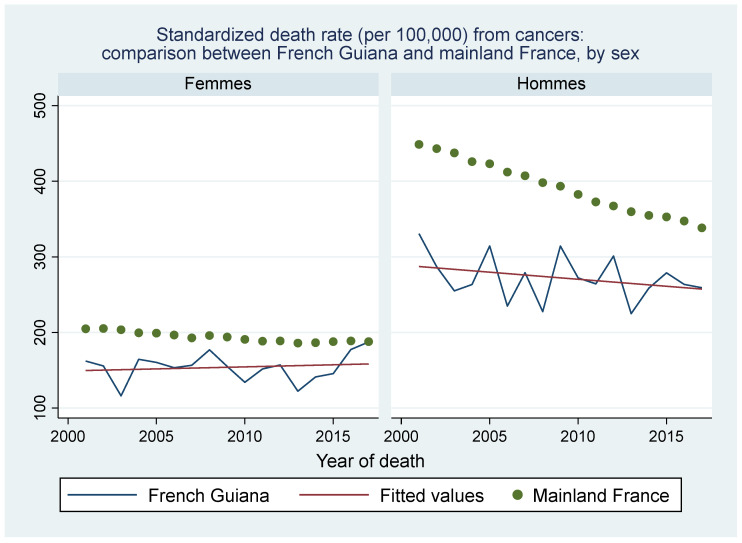
Standardized death rate from cancers: comparison between French Guiana and mainland France, by sex. Green dots represent mainland France, the blue line represents French Guiana and the red line is the fitted line for French Guiana.

Figure 5 shows that deaths from cancer in French Guiana remained stable and lower than in mainland France where male deaths from cancer were highest but declined substantially.

**Figure 6:**
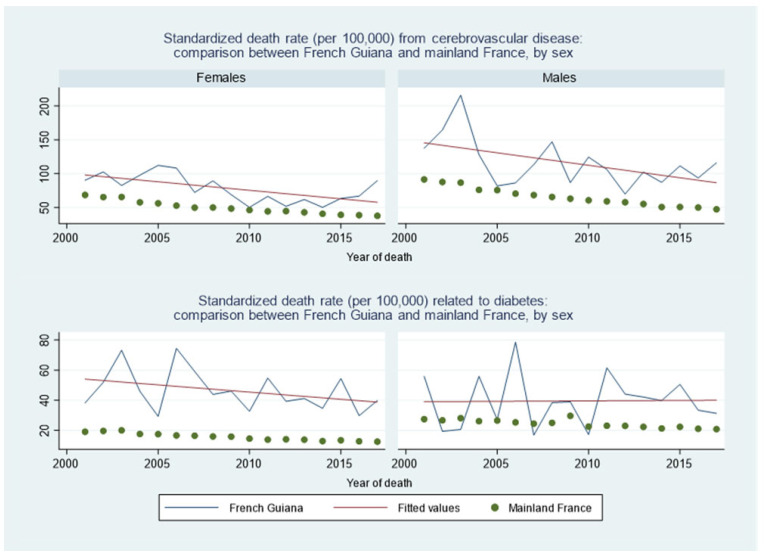
Standardized death rate from cerebrovascular disease: comparison between French Guiana and mainland France, by sex. Green dots represent mainland France, the blue line represents French Guiana and the red line is the fitted line for French Guiana.

For circulatory diseases, the death rate declined both in French Guiana and France. The top two plots in Figure 6 show that, although in regular decline, cerebrovascular diseases were more frequent in French Guiana than in France, especially in males. The two bottom graphs of Figure 6 show that the excess of mortality linked to diabetes was substantially greater among females than among males. However, mortality from diabetes apparently tended to decline in females but not in males.

**Figure 7:**
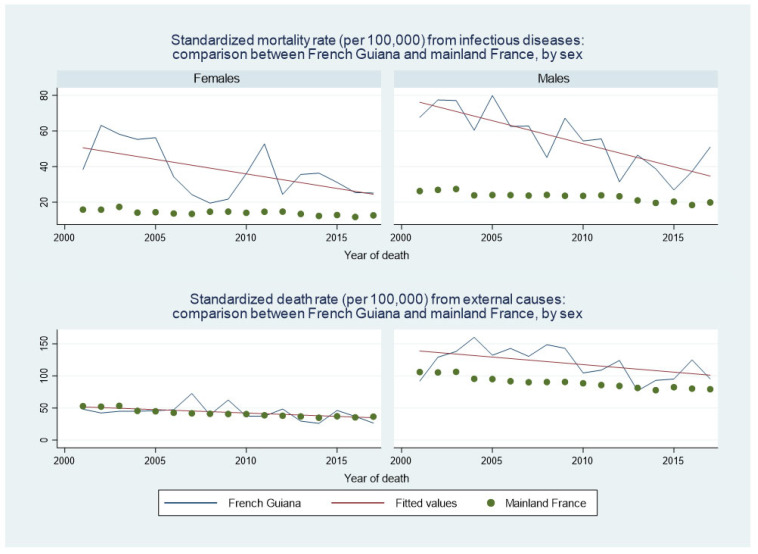
Standardized death rate from infectious disease: comparison between French Guiana and mainland France, by sex. Green dots represent mainland France, the blue line represents French Guiana and the red line is the fitted line for French Guiana.

Although deaths from infectious diseases were greater in French Guiana than in mainland France, Figure 7 shows the rapid decline in infectious causes of death in French Guiana both in females (left) and males (right). Figure 7 also shows that for external causes, there was a downward trend in males and in females, with no difference between French Guianese and French females, which contrasted with the consistent excess in mortality among French Guianese males.

**Figure 8:**
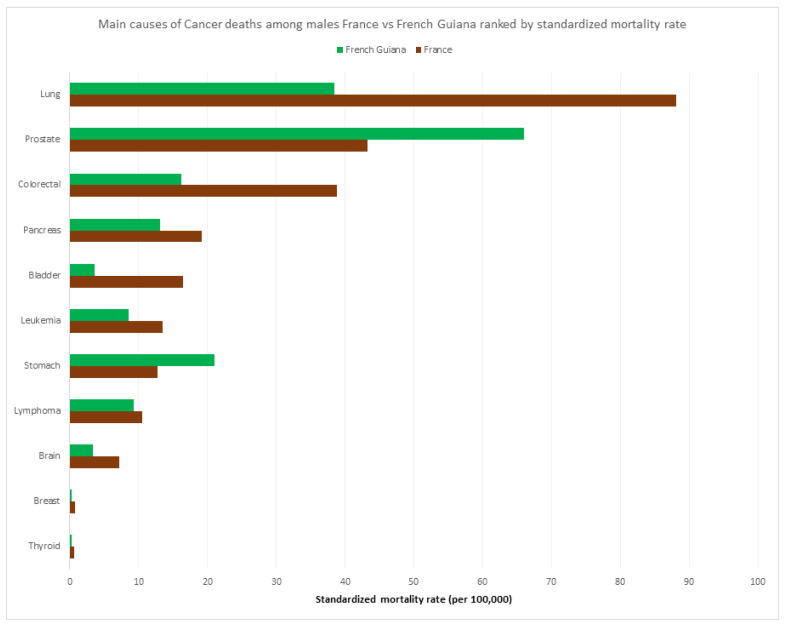
Main causes of cancers among males France vs. French Guiana ranked by standardized mortality rate.

**Figure 9:**
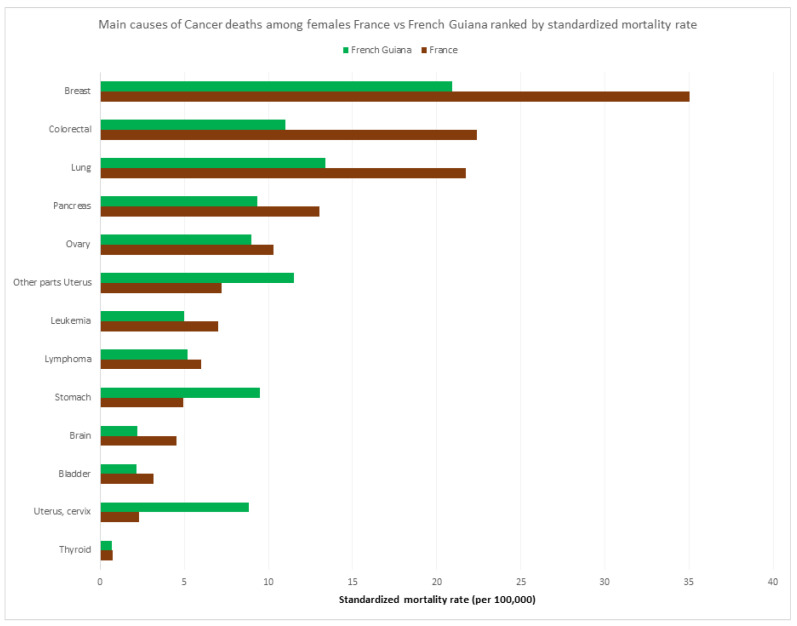
Main causes of cancers among females France vs. French Guiana ranked by standardized mortality rate.

Figure 8 and Figure 9 show the respective ranking of the standardized death rate by cancer for males and females. Apart from the overall excess cancer deaths in males versus females (Figure 5), and in mainland France versus French Guiana, the most striking specificities in French Guiana for males were the excess burden of prostate cancer and stomach cancer relative to mainland France, and for women, the excess burden of uterus cancer (cervix and other parts) and stomach cancer. Among women, two of the main cancers were related to an infectious agent (HPV, and *H. pylori*).

### 3.1. Life Expectancy at Birth

**Figure 10:**
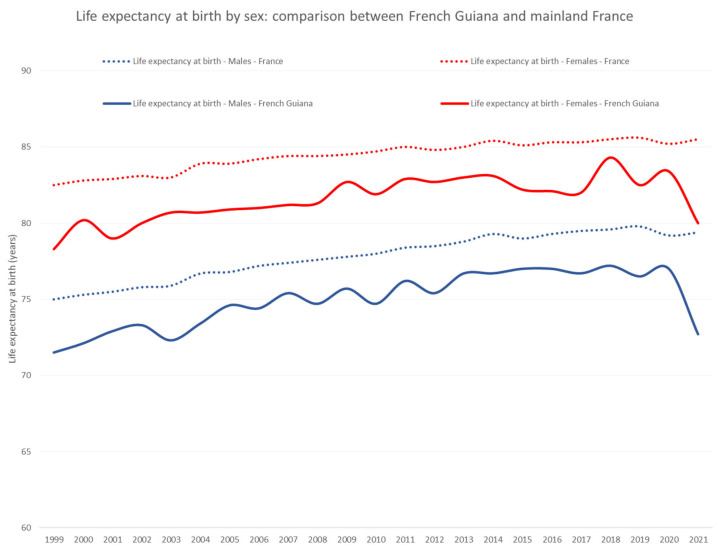
Life expectancy at birth by sex: comparison between French Guiana and mainland France. The dotted lines represent mainland France and the solid lines represent French Guiana. Females are in red and males in blue.

Life expectancy at birth grew until 2020, but there was a steady gap for females (median = 3 years) in French Guiana, and a 2-year gap for males (median = 2.6 years) (Figure 10. However, in 2021, COVID-19 and low vaccine uptake were associated with a drop of life expectancy at birth in French Guiana relative to mainland France: the difference was 5.5 years for females and 6.7 years for males.

### 3.2. At 20, 40, and 60 Years

**Figure 11:**
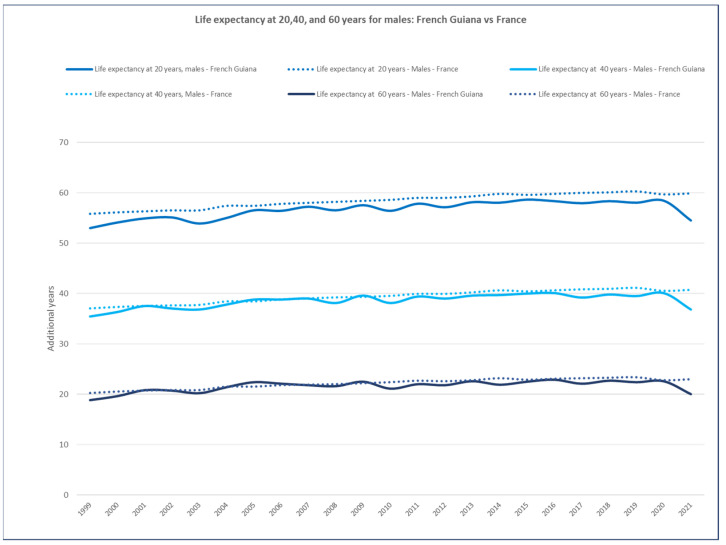
Life expectancy at 20, 40, and 60 years for male: French Guiana vs. France. The dotted lines represent mainland France and the solid lines represent French Guiana.

Excluding 2021 (massively impacted by COVID-19), when looking at life expectancy at ages 20, 40, and 60 years (Figure 11), it appeared that in males, at 40 and 60 years, life expectancy was slightly lower (*p* = 0.02) at 40 or on par at 60 years (*p* = 0.11) with that in mainland France. However, at 20 years, it was clearly lower than in mainland France.

**Figure 12:**
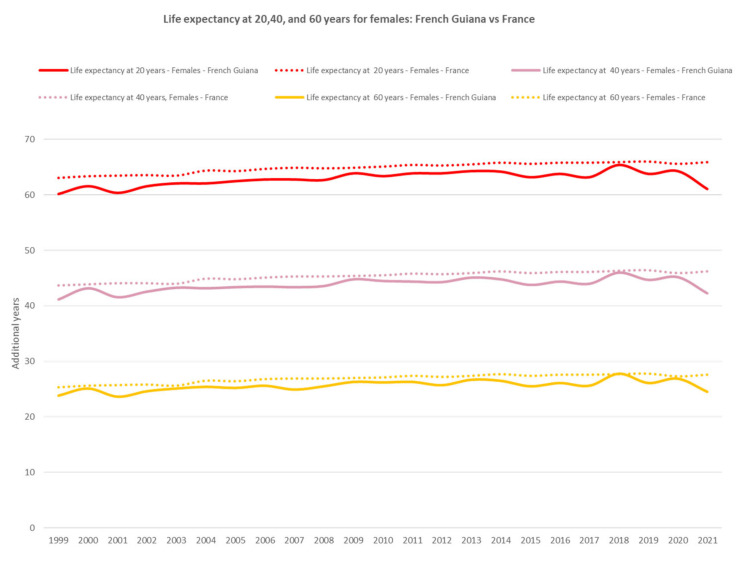
Life expectancy at 20, 40, and 60 years for females: French Guiana vs. France. The dotted lines represent mainland France and the solid lines represent French Guiana.

By contrast, among females, life expectancy at 20, 40, and 60 years was consistently lower than that observed in mainland France (Figure 12).

### 3.3. Poor versus Rich

When applying the INSEE tool using income levels close to the median income of French Guiana, there was a 3.8 year-difference for women and a 5.8 year-difference for men between the 1000 euro per month group and the 2000 euro per month group [16]. Given that 23% of persons live with less than 550 euros per month, the INSEE tool shows that the estimated difference in life expectancy at birth between those living with 500 euros per month versus those living with 2000 euros per month was 6.6 years for women and 9.3 years for men.

## 4. Discussion

The complex picture that emerges from these data has different layers: first, when comparing with mainland France, life expectancy at birth in French Guiana was significantly shorter both in males and females; unsurprisingly, the standardized death rate of males in French Guiana was substantially greater than that of women, and their life expectancy at birth was significantly shorter. Life expectancy at 20 years, which allows to remove the effect of the greater child mortality in French Guiana [17], was also shorter in French Guiana for males and females, perhaps reflecting the burden of external causes (accidents, homicides, drowning…) and AIDS in males and perhaps metabolic diseases in women—mostly diabetes. More surprisingly, the differences between mainland France and French Guiana regarding life expectancy at 40 and 60 years was mainly found among females, while males in French Guiana had a slightly lower or similar life expectancy at 40 and 60 years than in mainland France. 

The relative weight of major risk factors in French Guiana—the French Guianese exposome—was quite different from mainland France, which presumably largely explains why the age-standardized cancer deaths in French Guiana were so much lower than in mainland France. Smoking and alcohol consumption were much less frequent in French Guiana than in France [18]; hence, daily smoking was reported in 16% of males in French Guiana vs. 32% in mainland France, and for females, 8% in French Guiana vs. 24% in mainland France; for alcohol daily consumption was reported in 4.8% in French Guiana versus 9.7% in mainland France [18]. By contrast, in French Guiana, external causes (accidents, homicides, and drownings, notably) were more frequent, notably among young males (there was no difference between French Guiana and France for females (Figure 7)). A noteworthy point was that suicides were the fifth cause of premature death in mainland France, whereas it was the 9th in French Guiana. This was somewhat counterintuitive because the Amerindian communities living in the isolated interior of French Guiana have a very high incidence of suicide [19,20], the “macro” observation here reflects the fact that most of the population lives along the coastline where suicide incidence is lower.

In French Guiana, infectious diseases, notably AIDS, were much more frequent in both males and females, and hypertension, obesity, and metabolic diseases were far more frequent in females [18]. Women in French Guiana are substantially more affected by overweight/obesity and type 2 diabetes [18]. Although women consume more health services, they remain more affected by these major risk factors [21,22], and when exposed, they are more prone to cardiovascular risks than men [23]. The proportion of obese (body mass index > 30 kg/m^2^) was greater among French Guianese women (23%) than in women in mainland France (12%) and French Guianese men (13% versus 12% in mainland France) [18]. Being overweight/obese in French Guiana was significantly associated with precariousness, being an immigrant, and having a mismatch between body image and obesity [14]. Although in French Guiana, women consulted more frequently than males in the past year (85% versus 71%), they were also significantly more likely to have renounced care for both financial reasons and lack of time [11]. Finally, in the health barometer survey, a lower proportion of females than males declared that they thought their health was good (58% versus 72%, respectively), a finding that is regularly observed [24]. Beyond the problem of obesity in French Guiana, there is often the hidden problem of malnutrition. Hence, among pregnant women, while 50% were overweight or obese, 81% had at least one micronutrient deficiency, and 46% had two deficiencies [25]. Food insecurity is relatively common in poor neighborhoods of French Guiana [26]. In this deprivation context, the high prevalence of obesity is not so surprising [27]. Furthermore, the high risk of metabolic and cardiovascular diseases in French Guiana may have links with epigenetic factors triggered by nutritional anomalies during pregnancy and early childhood [28,29]. Finally, in a population predominantly of African ancestry, the high prevalence of obesity, notably among women, raises the question of genetic predispositions for weight gain and insulin resistance early on—early hyperinsulinism and insulin resistance were shown in Black teens—and after pregnancy in women [30,31]. Disentangling genetic factors, epigenetic factors, social factors, and cultural factors is not easy, but there is indeed a convergence of causal factors that explain some of the differences seen in terms of life statistics.

Although this may reflect different mixes of risk factors between French Guiana and mainland France, or different exposomes, the similar life expectancies between males aged 40 and 60 years seems to suggest that, overall, the level of hospital care may not be the most salient difference. This is a possible argument against the general perception that much of the difference in life expectancy between France and French Guiana is attributable to an unjust lack of investment in hospital infrastructure [32,33].

To close the gap in life expectancy at birth between French Guiana and France and between males and females, strategic interventions must be deployed to alleviate the burden of the main causes of early death, many of which are sensitive to primary health care. Unfortunately, much of the political debate on health is polarized by the hospital system and not on optimizing strategies that would impact early mortality. In French Guiana, the trend from 2001 to 2017 was generally improved, notably in some of the main causes of death—AIDS, cardiovascular diseases, even external causes.

The present study had limitations that must be taken in consideration for interpreting the present results. First, the quality of death certificates was always imperfect and may lead to some imprecision both in French Guiana and mainland France. Some of the present discussion relied on correlations, which may not necessarily reflect causal relationships. The data from French Guiana originate in a small population (circa 300,000 persons), which led to wide yearly fluctuations and jagged lines; our trend line aimed to smoothen the trend but, again, the secular pattern may not have been captured by a line fitted using linear regression. The present results focused on death but provided no data on disability, which is often used in establishing the burden of diseases. Finally, French Guiana is a highly contrasted territory where crude measures can be misleading. It is likely that the richer half of the population living in French Guiana has a similar life expectancy than in mainland France. However, the median income in French Guiana is only half of that of France and food is more expensive in French Guiana; therefore, large within territory differences in life statistics are expectable. Using the INSEE tool to compute life expectancy at birth by income strata, there was a 3.8 year-difference for women and a 5.8 year-difference for men between the 1000-euros-per-month group and the 2000-euros-per-month group [16]. Given that a substantial proportion of persons live with less than 500 euros per month in French Guiana, the INSEE tool showed that the estimated difference in life expectancy at birth between those living with 500 euros per month versus those living with 2000 euros per month was 6.6 years for women and 9.3 years for men. Although these approximations illustrating the impact of poverty were interesting, such comparisons using national tools computing national probabilities of survival at different ages did not account for the major differences between the conditions in French Guiana and mainland France.

## 5. Conclusions

The present study showed the quantitative and qualitative differences in the main life expectancy and mortality indicators for males and females. Although, as elsewhere, life expectancy at birth was lower in males than in females, at ages 40 and 60, there was a greater difference between French Guiana and mainland France, i.e., a difference that may reflect the burden of obesity and diabetes in the French Guianese female population. Such differences illustrate the complex interplay between genetic, epigenetic, social, and cultural factors present in French Guiana.

## Data Availability

The data are public data that are available to all.
